# Inequities in Opioid Administration by Race and Ethnicity for Hospitalized Patients With and Without Substance Use Disorders

**DOI:** 10.1007/s11606-025-09514-6

**Published:** 2025-04-25

**Authors:** Aksharananda Rambachan, Margaret C. Fang

**Affiliations:** Division of Hospital Medicine, Department of Medicine, University of California, San Francisco, San Francisco, CA USA

**Keywords:** Health equity, Pain management, Substance use disorders, Hospital medicine

## Abstract

**Background:**

Adequate pain management is challenging in patients with substance use disorders, particularly those from racial/ethnic minority groups who face intersecting biases.

**Objective:**

To investigate inequities in pain management for racial/ethnic minority groups with and without concurrent substance use disorders.

**Design:**

Retrospective cohort study from 2021 to 2022 on an acute care general medicine service at UCSF Medical Center.

**Participants:**

All adults ≥ 18 years old.

**Exposures:**

Primary exposure was the patient’s self-identified race/ethnicity (Asian, Black or African American, Latino, Multi-Race/Ethnicity, Native American or Alaska Native, Native Hawaiian or Pacific Islander, Southwest Asian or North African, White, Other, and Unknown/Declined).

**Main Outcome and Measures:**

The primary outcome was average daily inpatient opioids received (morphine milligram equivalents, MME). Multivariable negative binomial regression assessed the relationship between self-reported race/ethnicity and opioid administration, adjusting for demographics, clinical factors, substance use disorders, and pain characteristics. The subgroup analyses focused on patients with substance use disorders and on patients without any buprenorphine or methadone prescriptions.

**Key Results:**

In the overall cohort of 13,058 hospitalizations (mean age 62.7 years, 51.2% male, 31.3% with substance use disorder), patients from racial/ethnic minority groups received significantly fewer opioids than White patients in adjusted analyses: Asian (− 61.3 MME/day), Black (− 44.9 MME/day), Latino (− 48.8 MME/day), Native American/Alaska Native (− 80.4 MME/day), and Native Hawaiian/Pacific Islander (− 72.9 MME/day). Similar, significant disparities were present in both subgroups. Notably, in the substance use disorder-only subgroup (*n* = 4446), larger disparities persisted for Asian (− 124.4 MME/day), Black (− 68.7 MME/day), and Latino (− 110.8 MME/day) patients compared to White patients.

**Conclusions:**

Substantial racial/ethnic inequities in inpatient opioid prescribing for pain control were observed, particularly among patients with concurrent substance use disorders. These findings highlight the need for interventions promoting equitable, culturally competent pain management for marginalized populations facing intersecting biases and stigma.

**Supplementary Information:**

The online version contains supplementary material available at 10.1007/s11606-025-09514-6.

## INTRODUCTION

Effective and equitable pain management for patients with substance use disorders from minoritized groups presents unique challenges.^1–3^ Patients with substance use disorders, particularly opioid use disorder, often have higher opioid tolerance and hyperalgesia and may concurrently be withdrawing during hospitalization.^4^ Rates of certain substance use disorders for minoritized patients vary, and there is demonstrated literature showing inequities in pain management for patients from minoritized groups.^5–8^ Prior research has demonstrated that there is mutual distrust between physicians and patients from both of these groups, including a belief that clinicians do not take the pain reported by a patient seriously.^5,8,9^ Clinicians may hesitate to prescribe necessary doses of opioids to treat pain in these patient groups because of concerns about drug-seeking behavior, diversion, elopement risk, and respiratory depression.^1,10^ There is a major gap in understanding whether and to what extent inequities in pain management exist for inpatients with substance use disorders who identify as a racial or ethnic minority.^11^

We hypothesize that patients from minoritized racial/ethnic groups experience disparate pain management during hospitalization, after accounting for the presence of a substance use disorder. To test this hypothesis, we studied the association between racial/ethnic category and inpatient opioids received, after controlling for demographic and clinical factors, the presence of a substance use disorder, and pain characteristics. This study helps to characterize opioid prescribing for a challenging clinical population where equitable care is an increasing focus.

## METHODS

### Study Population

This retrospective cohort study included adult hospitalizations (≥ 18 years old) from January 1, 2021, to December 31, 2022, discharged from the general medicine service at the University of California, San Francisco Medical Center at Parnassus Heights, a 785-bed academic medical center that serves a diverse patient population. All data was collected from Epic,^12^ our medical system’s electronic health record (EHR), and Clarity,^13^ the relational database that extracts and stores inpatient Epic data. We only included patient hospitalizations with complete pain assessment data using self-reported pain assessments. Patient hospitalizations were additionally excluded if the patient spent time in the intensive care unit or if they received inpatient intensive comfort-focused or hospice care because these patients have different pain requirements and have their care often managed by specialists. The UCSF Institutional Review Board for Human Subjects Research approved this study with a waiver of informed consent.

### Predictor/Exposure

The primary predictor was the patient’s self-reported race/ethnicity. Consistent with updated US Census, NIH reporting,^14^ and institutional standards, we included the following minority race/ethnicity categories: Asian, Black or African American, Latino, Multi-Race/Ethnicity, Native American or Alaska Native, Native Hawaiian or Pacific Islander, Southwest Asian or North African, Other, and Unknown/Declined, with White as a comparison group. These racial/ethnic group identities are socially, not genetically defined.^15^ These categorizations are used as a proxy for how race and ethnicity intersect with equity in healthcare and may help guide future research on mechanisms that create inequity, including racism.^16^

### Outcome

The primary outcome was the average daily inpatient opioids received during the patient’s hospitalization, measured by morphine milligram equivalents (MMEs). This is calculated by our Division’s Data Core using standardized conversions of opioid medications from the EHR.^17^

### Covariates

All analyses were adjusted with demographic, clinical, substance-use, and pain-related variables. Demographic variables included patient age, self-reported sex (male, female, or non-binary/other), limited English proficiency status (defined as having a preferred language for healthcare other than English and requiring a medical interpreter), and insurance status (Medicare, Medi-cal, or Private/Other). Clinical variables included the Elixhauser comorbidity index as a marker of clinical complexity and length of stay.^18^ Substance-use-related variables included having a billing diagnosis of any substance-use disorder using International Classification of Diseases (ICD)− 10 codes.^19^ These ICD- 10 codes were manually selected by the authors to reflect whether a patient had a billing diagnosis that would best reflect a clinical diagnosis of a substance use disorder (Supplemental Table 2). We also included whether a patient had an existing prescription for Medication for Opioid Use Disorder (MOUD) on admission (methadone or buprenorphine), based on admission medication reconciliation, which includes confirmation with methadone clinic. Pain-related variables included the patient’s average self-reported pain score during their entire hospitalization, whether the patient was admitted with moderate/severe pain (defined as their first pain assessment being ≥ 6 on a 0–10 self-reported pain scale), whether a consult was placed for the pain or palliative medicine service, prior to admission opioid prescription (i.e. oxycodone, morphine, etc.), and the average daily milligrams of acetaminophen and ibuprofen administered to the patient. At our institution, the pain service is staffed by anesthesia, the palliative medicine is staffed by palliative medicine physicians, and either service may be consulted for pain-related issues. The average self-reported pain score was calculated as the mean of all Numeric Rating Scale, Verbal Descriptor Scale, and FACES Pain Scale-Revised Scores standardized to a 0–10 scale, with higher numbers reflective of worse pain. Nursing pain assessments are performed throughout a patient’s hospitalization: on admission, on unit transfers, before, during, and after procedures or analgesic administration, and with routine vital sign checks. These data are inputted by nurses into EHR flowsheets.^6^

### Statistical Analysis

All analyses were done using Stata software v.18. To assess for differences in inpatient pain management, we first calculated the unadjusted daily MMEs across race/ethnicity and all the other covariates. For the adjusted analysis, we used multivariable negative binomial regression to account for the heavily dispersed distribution of daily MMEs. The models were adjusted for all demographic, clinical, substance use, and pain-related variables, with clustering by patient medical record number to account for multiple hospitalizations for a given patient during the study period. All hypothesis tests were evaluated at *α* = 0.05. We prespecified the interaction between race/ethnicity and substance use disorder and utilized omnibus testing. If the interaction was not significant, we refit the model with main effects only. White race was used as the reference category. Results from the negative binomial regression were reported using average marginal effects (AMEs), which describes the average difference in average daily inpatient MMEs between the comparison and reference race/ethnicity categories.^20^

### Subgroup Analyses

We performed two subgroup analyses. First, we repeated the above analysis on only patients with an ICD- 10-defined substance use disorder. In this subgroup, we reported the frequency of each specific substance use disorder and performed an adjusted analysis using negative binomial regression. The purpose of this subgroup analysis was to further isolate the effect of race/ethnicity on inpatient opioid administration by minimizing confounding and focusing only on those with a defined substance use disorder. Second, we performed the above analysis excluding all patients who were on either methadone or buprenorphine prior to admission and all the patients who received methadone or buprenorphine during admission. The purpose of this subgroup was to minimize confounding by medications that can be used to treat either OUD or pain and potential variation in MOUD prescribing rates among different racial/ethnic groups. This subgroup did not include any methadone or buprenorphine calculated in the outcome variable of MMEs.

## Results

**Figure 1:**
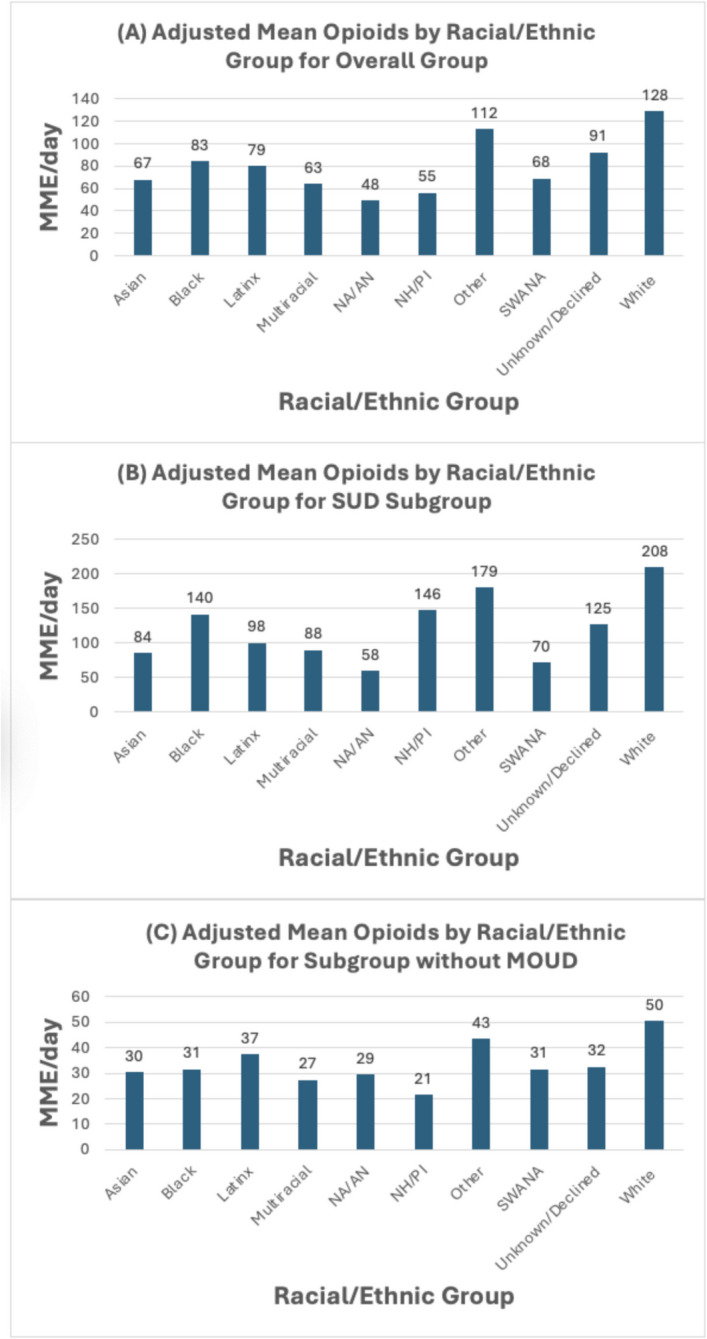
**A**–**C** Adjusted mean opioids for the overall cohort, SUD subgroup, and subgroup without MOUD. NA/AN, Native American or Alaska Native; NH/PI, Native Hawaiian or Pacific Islander; SWANA, Southwest Asian or North African

The study included 9102 patients across 13,058 unique hospitalizations from January 1, 2021, to December 31, 2022, discharged from the general medicine service. The cohort had a mean age of 62.7 years (standard deviation (SD) 19.0) and was 51.2% male. The racial/ethnic distribution of the overall cohort was 43.3% White, 23.2% Asian, 13.0% Latino, 12.3% Black or African American, and the remaining groups made up 8.4% (Fig. 1). At the individual patient level, 31.3% of patients had a substance use disorder diagnosis, 4.2% were prescribed MOUD prior to admission, 26.3% were prescribed opioids prior to admission, and 31.8% were admitted with moderate or severe pain. Rates of MOUD prior to admission varied across racial and ethnic groups (White, 5.6%; Black, 6.5%; Asian, 1.4%; and Latino, 2.6%). The mean hospital length of stay was 6.8 days (SD 13.7) (Table 1, see Supplemental Table 1 for data across all race/ethnicity categories).

**Table 1:** Baseline Characteristics for Medical Patients Hospitalized Between 2021 and 2022, No. % (*N* = 9102 Patients Across 13,058 Hospitalizations*)

Variable		By race/ethnicity^‡^			
	Overall *n* = 9102	White *n* = 3939	Asian *n* = 2110	Latino *n* = 1181	Black *n* = 1116
Selected demographic and hospitalization-related variables					
Age in years, mean (SD)	62.7 (19.0)	63.5 (18.1)	69.3 (18.6)	54.7 (19.9)	58.9 (17.0)
Length of Stay in days, mean (SD)	6.8 (13.7)	6.5 (11.1)	6.0 (8.1)	7.2 (16.9)	8.3 (18.2)
Comorbidity index, mean (SD)	9.5 (11.2)	9.2 (11.2)	10.9 (11.1)	9.6 (11.8)	9.0 (11.2)
Sex					
Female	4432 (48.7%)	1788 (45.4%)	1112 (52.7%)	633 (53.6%)	535 (47.9%)
Male	4656 (51.2%)	2143 (54.5%)	997 (47.3%)	548 (46.4%)	580 (52.0%)
Nonbinary/Other	14 (0.2%)	8 (0.2%)	1 (0.1%)	0 (0.0%)	1 (0.1%)
Limited English proficiency					
Yes	1885 (20.7%)	211 (5.4%)	1100 (52.1%)	412 (34.9%)	14 (1.3%)
No	7217 (79.3%)	3728 (94.6%)	1010 (47.9%)	769 (65.1%)	1102 (98.8%)
Substance use and pain-related variables					
Presence of substance use disorder	2846 (31.3%)	1378 (35.0%)	282 (13.4%)	347 (29.4%)	617 (55.3%)
Prescribed MOUD^†^, prior to admission	386 (4.2%)	223 (5.7%)	31 (1.5%)	32 (2.7%)	76 (6.8%)
Prescribed opioids prior to admission	2389 (26.3%)	1116 (28.3%)	395 (18.7%)	333 (28.2%)	348 (31.2%)
Received pain or palliative care consultation	379 (4.2%)	170 (4.3%)	63 (3.0%)	50 (4.2%)	56 (5.0%)
Pain score during hospitalization, mean (SD)	2.1 (2.2)	2.2 (2.2)	1.3 (1.6)	2.3 (2.2)	2.7 (2.5)
Admitted with moderate/severe pain	2850 (31.3%)	1192 (30.3%)	481 (22.8%)	471 (39.9%)	452 (40.5%)
Average daily acetaminophen in mg, mean (SD)	840.6 (932.1)	862.7 (950.7)	670 (846.6)	933.3 (951.5)	950.5 (953.7)
Average daily ibuprofen in mg, mean (SD)	47.1 (206.8)	53.1 (217.1)	28.1 (158.9)	54.5 (231.7)	51.8 (217.0)

^*^The results in Table 1 are calculated at the individual patient level (*N* = 9102) for the first hospitalization for each given patient

^†^*MOUD* medication for opioid use disorder

^‡^The four most common race/ethnicity groups are displayed (comprising 91.8% of patients), full results available in Supplemental Table 1

### Primary Analysis

Unadjusted and adjusted analyses examining the relationship between race/ethnicity and average daily inpatient opioid administration in morphine milligram equivalents (MME) are presented in Table 2. In the unadjusted analysis, significant differences were found across racial/ethnic groups (*p* < 0.001), with Black or African American patients receiving the highest mean MME/day of 81.3 (SD 192.7) and Asian patients the lowest at 12.9/day (SD 65.3) (Table 2).

**Table 2:** Unadjusted and Adjusted MME/Day* Regression Results for 13,058 Hospitalizations

Variable	Unadjusted MMEs, mean (SD)	*p*-value	Adjusted MMEs, mean (95% CI)	*p*-value
Race/ethnicity				
White	57.9 (168.8)	< 0.001	128.1 (105.9–150.4)	Ref
Asian	12.9 (65.3)		66.8 (50.1–83.6)	< 0.001
Latino	39.8 (109.1)		79.3 (62.6–96.1)	< 0.001
Black or African American	81.3 (192.7)		83.2 (65.1–101.3)	< 0.001
Multi-Race/Ethnicity	31.1 (65.5)		63.3 (40.9–85.8)	< 0.001
Other	43.2 (124.7)		112.2 (61.4–163.0)	0.530
Southwest Asian or North African	19.7 (57.5)		68.0 (40.5–95.5)	< 0.001
Native Hawaiian or Pacific Islander	31.5 (103.0)		55.3 (32.0–78.5)	< 0.001
Unknown/Declined	25.8 (86.1)		91.0 (31.6–150.3)	0.213
Native American or Alaska Native	80.9 (113.3)		47.7 (9.3–86.1)	< 0.001
Limited English proficiency				
Yes	12.9 (68.6)	< 0.001	87.5 (67.2–107.9)	0.229
No	55.2 (156.5)		97.9 (83.0–112.8)	
Sex				
Male	45.9 (143.0)	0.848	108.0 (89.6–126.4)	Ref
Female	47.0 (144.9)		89.0 (74.6–103.4)	0.007
Nonbinary/other	58.7 (114.7)		66.6 (15.7–117.5)	0.117
Insurance status				
Medicare	27.8 (95.5)	< 0.001	84.7 (68.5–100.9)	Ref
Medical	91.3 (222.9)		110.2 (90.1–130.4)	0.019
Private/other	41.5 (116.4)		84.0 (68.0–100.1)	0.931
Pain/palliative care consultation				
Yes	240.3 (363.9)	< 0.001	138.9 (110.5–167.3)	< 0.001
No	36.0 (112.0)		84.4 (72.0–96.7)	
Presence of substance use disorder				
Yes	92.3 (203.2)	< 0.001	107.9 (91.6–124.2)	< 0.001
No	22.8 (91.7)		71.7 (57.4–86.0)	
Prescribed MOUD prior to admission				
Yes	311.3 (365.5)	< 0.001	663.7 (509.9–817.6)	< 0.001
No	31.8 (101.9)		45.1 (40.4–49.7)	
Prescribed opioids prior to admission				
Yes	72.9 (154.8)	< 0.001	204.1 (163.0–245.2)	< 0.001
No	35.7 (137.6)		78.2 (64.4–92.0)	
Admitted with moderate/severe pain				
Yes	96.2 (205.9)	< 0.001	97.4 (82.5–112.3)	0.933
No	21.6 (89.4)		97.9 (80.0–115.9)	
Continuous covariates^†^				
Age	− 1.50 (− 1.62 to − 1.37)	< 0.001	− 2.4 (− 3.1 to − 1.8)	< 0.001
Elixhauser comorbidity score	− 0.44 (− 0.65 to − 0.22)	< 0.001	0.2 (− 0.4 to 0.7)	0.562
Average acetaminophen/day	0.03 (0.03–0.04)	< 0.001	0.24 (0.17–0.03)	< 0.001
Average ibuprofen/day	0.08 (0.06–0.09)	< 0.001	− 0.03 (− 0.05 to 0.0)	< 0.001
Average pain score/hospitalization	27.3 (26.3–28.2)	< 0.001	− 0.5 (− 12.7 to 11.6)	0.933
Length of stay	0.84 (0.65–1.04)	< 0.001	− 0.8 (− 1.3 to − 0.4)	0.001

^*^Adjusted MME/day calculated using average marginal effects. Regression used multivariable negative binomial regression with robust clustering by medical record number. Omnibus interaction between race/ethnicity and substance use disorder diagnosis was non-significant (df = 9, chi^2^ = 14.1, *p* * 0.119) and thus not included in the final model

^†^The unadjusted column coefficients are calculated based on unadjusted linear regression with 95% confidence intervals

For the adjusted analysis, after considering demographic factors, clinical variables, substance use, pain characteristics, and accounting for clustering of hospitalizations by patient, significant differences in opioid administration were found across racial/ethnic groups. Every defined racial/ethnic minority group, except for “Other” and “Unknown/Declined” received significantly fewer opioids compared to White patients. The largest racial/ethnic minority groups all received fewer adjusted opioids, including Asian patients (− 61.3 MME/day, 95% confidence interval (CI) − 79.4 to − 43.2, *p* < 0.001), Black patients (− 44.9 MME/day, 95% CI − 68.9 to − 21.0, *p* < 0.001), and Latino patients (− 48.8 MME/day, 95% CI − 68.6 to − 29.0, *p* < 0.001). The largest effect sizes were found for Native American/Alaska Native patients (− 80.4 MME/day, 95% CI − 121.6 to − 39.2, *p* < 0.001) and Native Hawaiian/Pacific Islander patients (− 72.9 MME/day, 95% CI − 99.5 to − 26.3, *p* < 0.001). Several other factors were associated with higher opioid administration, including the presence of a substance use disorder diagnosis, being prescribed MOUD or opioids prior to administration, and receiving an inpatient pain or palliative medicine consultation. Patients with higher levels of pain on admission did not receive higher levels of opioids compared to those without higher levels of pain (Table 2).

### Subgroup Analysis 1: Patients with SUD

Compared to the overall cohort, this specific subgroup of patients with SUD (*n* = 2846 patients across 4446 hospitalizations) had a higher proportion of Black or African-American and male patients and fewer Asian patients (Table 3). The most common substance-related disorders were nicotine/tobacco, alcohol, and opioids. For the adjusted analysis of the subgroup, the overall findings were similar, with most racial/ethnic minority groups receiving fewer adjusted opioids compared to White patients. For the largest racial/ethnic minority groups, Asian (− 124.4 MME/day, 95% CI − 168.3 to − 80.6, *p* < 0.001), Black (− 68.7 MME/day, 95% CI − 120.9 to − 16.6, *p* < 0.001), and Latino patients (− 110.8 MME/day, 95% CI − 155.8 to − 65.9, *p* < 0.001) all received fewer opioids than White patients. These effect sizes were all larger than in the overall analysis (Table 4).

**Table 3:** Baseline Characteristics for Subgroup of Medical Patients Hospitalized Between 2021 and 2022 Who Had a SUD, No. % (*N* = 2846 Patients Across 4446 Hospitalizations)

Variable		By race/ethnicity**			
	Overall	White	Asian	Latino	Black
Selected demographic and hospitalization-related variables					
Age in years, mean (SD)	56.3 (16.7)	57.6 (16.7)	60.9 (17.6)	50.7 (17.5)	55.5 (15.0)
Length of stay in days, mean (SD)	7.7 (18.1)	7.3 (14.4)	6.0 (8.5)	8.6 (25.7)	9.0 (21.8)
Comorbidity index, mean (SD)	8.5 (11.7)	8.1 (11.5)	10.3 (11.5)	8.5 (12.1)	8.8 (11.8)
Sex					
Female	1066 (37.5%)	519 (37.7%)	83 (29.4%)	125 (36.0%)	251 (40.7%)
Male	1773 (62.3%)	854 (61.2%)	199 (70.6%)	222 (64.0%)	365 (59.2%)
Nonbinary/other	5 (0.4%)	5 (0.4%)	0 (0.0%)	0 (0.0%)	0 (0.0%)
Limited English proficiency					
Yes	254 (8.9%)	30 (2.2%)	114 (40.4%)	75 (21.6%)	5 (0.8%)
No	2592 (91.1%)	1348 (97.8%)	168 (59.6%)	272 (78.4%)	612 (99.2%)
Substance use disorder					
Alcohol-related disorder	1019 (35.8%)	538 (39.0%)	56 (19.9%)	154 (44.4%)	203 (32.9%)
Cannabis-related disorder	185 (6.5%)	77 (5.6%)	12 (4.3%)	19 (5.5%)	61 (9.9%)
Cocaine-related disorder	359 (12.5%)	119 (8.6%)	15 (5.0%)	28 (8.0%)	177 (28.7%)
Hallucinogen-related disorder	14 (0.5%)	7 (0.5%)	0 (0.0%)	5 (1.4%)	0 (0.0%)
Inhalant-related disorder	3 (0.1%)	2 (0.2%)	0 (0.0%)	0 (0.0%)	1 (0.2%)
Nicotine/tobacco-related disorder	1753 (61.6%)	795 (57.7%)	199 (70.6%)	182 (52.5%)	424 (68.7%)
Opioid-related disorder	695 (24.4%)	378 (27.4%)	30 (10.6%)	65 (18.7%)	180 (29.9%)
Sedative, anxiolytic, or hypnotic-related disorder	145 (5.1%)	102 (7.4%)	8 (2.8%)	9 (2.6%)	14 (2.3%)
Stimulants or PCP-related disorder	529 (18.6%)	266 (19.3%)	30 (10.6%)	68 (19.6%)	136 (22.0%)
Presence of multiple substance, other, or unknown substance-related disorder	746 (26.2%)	382 (27.7%)	43 (15.3%)	71 (20.5%)	202 (32.7%)
Average # of substance use disorders, mean (SD)	1.9 (1.3)	1.9 (1.3)	1.4 (0.9)	1.7 (1.2)	2.3 (1.5)
Substance use and pain-related variables					
Prescribed MOUD* prior to admission	259 (9.1%)	156 (11.3%)	11 (3.9%)	18 (5.2%)	57 (9.2%)
Prescribed opioids prior to admission	793 (27.9%)	387 (28.1%)	62 (22.0%)	93 (26.8%)	197 (31.2%)
Received pain or palliative care consultation	128 (4.5%)	67 (4.9%)	9 (3.2%)	15 (4.3%)	25 (4.1%)
Pain score during hospitalization, mean (SD)	2.8 (2.5)	2.9 (2.5)	1.6 (2.0)	2.9 (2.6)	3.2 (2.6)
Admitted with moderate/severe pain	1136 (39.9%)	544 (39.5%)	80 (28.4%)	152 (43.8%)	279 (45.2%)
Average daily acetaminophen in mg, mean (SD)	923 (966)	913 (963)	710 (905)	916 (959)	1036 (995)
Average daily ibuprofen in mg, mean (SD)	62 (242)	73 (256)	33 (175)	60 (250)	53 (221)

^*^MOUD medication for opioid use disorder

^**^The four most common race/ethnicity groups are displayed (comprising 92.2% of patients)

**Table 4:** Adjusted MME/Day^a^ for SUD Subgroup

Variable	Adjusted MMEs, mean (95% CI)	*p*-value
Race/ethnicity		
White	208.3 (160.4–256.3)	Ref
Asian	83.9 (51.8–116.0)	< 0.001
Latino	97.5 (67.2–127.9)	< 0.001
Black or African American	139.6 (104.4–174.8)	0.010
Multi-Race/Ethnicity	88.2 (31.8–144.5)	< 0.001
Other	179.1 (39.7–318.5)	0.675
Southwest Asian or North African	70.0 (18.9–121.0)	< 0.001
Native Hawaiian or Pacific Islander	146.2 (− 53.0 to 345.4)	0.543
Unknown/Declined	125.1 (− 25.6 to 275.7)	0.277
Native American or Alaska Native	57.5 (18.9–96.1)	< 0.001
Limited English proficiency		
Yes	137.8 (75.3–200.2)	0.505
No	156.7 (126.8–186.5)	
Sex		
Male	174.7 (137.0–212.5)	Ref
Female	139.4 (108.2–170.6)	0.041
Nonbinary/other	40.3 (1.1–79.6)	< 0.001
Insurance status		
Medicare	165.7 (121.3–210.1)	Ref
Medical	167.1 (129.4–204.8)	0.956
Private/other	108.5 (79.2–137.8)	0.006
Pain/palliative care consultation		
Yes	190.8 (144.2–237.4)	0.016
No	145.6 (117.7–173.5)	
Prescribed MOUD prior to admission		
Yes	1000.3 (710.1–1290.5)	< 0.001
No	66.8 (58.5–75.0)	
Prescribed opioids prior to admission		
Yes	338.8 (247.4–430.2)	< 0.001
No	127.9 (99.2–156.5)	
Admitted with moderate/severe pain		
Yes	157.0 (126.3–187.6)	0.839
No	154.0 (117.4–190.6)	
Continuous covariates		
Age	− 4.3 (− 6.0 to − 2.6)	< 0.001
Elixhauser comorbidity score	− 0.6 (− 2.2 to 0.9)	0.409
Average acetaminophen/day	0.01 (0.0–0.03)	0.054
Average ibuprofen/day	0.0 (− 0.05 to 0.04)	0.893
Average pain score/hospitalization	56.6 (39.7–73.5)	< 0.001
Length of stay	− 0.4 (− 1.2 to 0.4)	0.325

^a^Adjusted MME/day calculated using average marginal effects. Regression used multivariable negative binomial regression with robust clustering by medical record number

### Subgroup Analysis 2: Excluding All Patients on Methadone and/or Buprenorphine

This subgroup included a total of 8612 patients across 12,153 hospitalizations. The cohort was very similar in composition to the overall cohort. For the adjusted analysis of this subgroup, our findings were similar to the overall analysis, where most racial/ethnic minority groups received fewer adjusted opioids compared to White patients. For the largest racial/ethnic minority groups, Asian (− 19.9 MME/day, 95% CI − 27.8 to − 11.9, *p* < 0.001), Black (− 19.3 MME/day, 95% CI − 27.9 to − 10.8, *p* < 0.001), and Latino (− 13.4 MME/day, 95% CI − 22.3 to − 4.62, *p* = 0.003) patients all received fewer opioids than White patients (Supplemental Table 3). Notably, average MMEs were lower in this subgroup compared to the overall cohort.

## Discussion

In this retrospective study of over 13,000 hospitalizations at an academic medical center, we found clinically meaningful racial/ethnic inequities in inpatient opioid administration for pain management after adjusting for substance use disorders, demographic factors, clinical variables, and pain characteristics. We found compounded inequity for patients with multiple marginalized identities (i.e., race/ethnicity minority, substance use disorder).

Consistent with our initial hypothesis, in our overall cohort, racial/ethnic minority groups received significantly fewer opioids compared to White patients. Large disparities were observed for Asian, Black, Latino, Native American/Alaska Native, Native Hawaiian/Pacific Islander, Southwest Asian/North African, and Multiracial patients. These findings persisted and were even more pronounced in the subgroup analysis restricted to patients with a substance use disorder diagnosis. We again found similar and substantial decreases in opioids received in the subgroup analysis that excluded all patients who received methadone or buprenorphine prior to or during admission. These are major findings to emphasize—even after controlling for key variables, including the presence of a substance use disorder, prior opioid and MOUD prescriptions, the average self-reported pain score, the presence of significant pain on admission, demographic and clinical characteristics, minoritized patients received significantly fewer opioids while admitted.

The effect sizes were not just statistically significant, but also clinically substantial. For context, a standard opioid dose prescribed by a physician is a 5 mg tablet of oxycodone, which is equivalent to 7.5 MMEs. In the subgroup, Asian patients received 124 fewer average daily MMEs than White patients, equivalent to 16 fewer tablets of oxycodone per day.

Our results are consistent with prior research demonstrating inequities for vulnerable groups in pain management across various healthcare settings, including those from racial/ethnic minority groups and those with a substance use disorder.^5–7^ However, this study extends those findings to the unique inpatient population of patients from racial/ethnic minority groups with a concurrent substance use disorder, a challenging clinical scenario where appropriate pain management is particularly complex.^1–3^ This study is also novel in that we studied and found inequities for often underreported racial/ethnic minority groups, including patients who identify as Native American/Alaska Native, Native Hawaiian/Pacific Islander, Southwest Asian/North African, and Multiracial.

There are several potential reasons for our findings, best understood by examination at the clinician, patient, and larger structural levels. At the clinician level, there is likely to be bias in providing care for patients with substance use disorders and those from minority racial/ethnic groups.^1,5,8–10^ These biases have been previously identified in the literature for both populations, and this patient population faces intersecting biases and stigma due to race/ethnicity and substance use. Clinicians may be particularly hesitant to prescribe opioids due to concerns about misuse, diversion, or exacerbating substance use disorders, despite evidence that undertreated pain can worsen outcomes.^21,22^

At the patient level, it is notable that Asian patients received the fewest opioids in both the overall and subgroup analyses. It is possible that there is variation across racial/ethnic groups in (1) the experience and expression of pain, (2) the ability or willingness to communicate a given pain level to a provider, and (3) the willingness to accept an opioid in general, or a higher dose of opioid pain medication for a given pain level.^23–26^ Despite these potential reasons, research in this area has still found that patient-related attitudinal concerns about opioids are more likely to be shaped by undertreatment, not as a cause of it.^27^

Between clinicians and patients, the role of communication is essential. Patients from minoritized racial groups are more likely to have limited English proficiency. While we did not find language status to be a significant predictor of opioid administration, there may still be cultural factors in terms of clinician-patient communication that impact overall pain assessment and management.^28^

At the systems level, there has been an increased national focus on opioid deprescribing.^29,30^ The study site was in San Francisco, a city particularly hit hard by the opioid epidemic with record overdose levels during the study period.^31^ This background is a likely factor in physician decisions on how aggressively to treat pain with opioids but does not fully explain the racial/ethnic variations in pain management.

There were several additional notable findings. In our models, the most significant predictors of receiving higher inpatient opioid doses, unsurprisingly, were having a prescription for MOUD and/or opioids prior to admission. These patients likely had higher opioid tolerance and the potential for hyperalgesia and concurrent withdrawal.^1,4^ Our subgroup analysis, which removed these patients, demonstrated lower doses of inpatient opioids but persistent racial/ethnic disparities. In the overall model, the average pain score was not associated with opioid administration, but this had a positive association in the subgroup models. As supported by the literature, reported pain is just one of many factors that influence the decision to prescribe opioids, particularly in those with concurrent substance use disorders.

There are limitations to consider. First, this was a single-center study where physicians at the study site likely systematically practice in different ways than other places, which limits our generalizability. Second, we used administrative billing codes to identify patients with substance use disorders, which could either underestimate or overestimate the true clinical prevalence, depending on the circumstance. Third, our capture of inpatient MMEs included methadone and buprenorphine for the overall model, which can be used for both pain control and for treatment for opioid use disorder. We are unable to parse out the indication for these medications in this dataset (for pain vs for OUD). Therefore, the second subgroup model, which eliminated all patients who received these medications prior to and during admission, was performed to minimize confounding from methadone and buprenorphine prescriptions.

Nonetheless, our findings are novel for the fields of general medicine, health equity, and substance use. Patients from minoritized groups who also have a substance use disorder are uniquely vulnerable to inequitable inpatient pain management. Future prospective studies including the specific indication for each opioid medication and clinician-based diagnoses of substance use disorders, and more granular analyses comparing different SUDs are needed to fully elucidate the mechanisms underlying these disparities. We also plan to examine the granular relationship between individual pain assessments and subsequent medication administration in various clinical scenarios across different patient cohorts, which would require multilevel time-series analysis to account for multiple prescribers, varying medication durations, and temporal relationships between pain scores and prescribing decisions. The consistency of all our findings across the overall cohort, the SUD subgroup, and the subgroup without buprenorphine/methadone highlights the importance of developing in-hospital interventions to promote equitable, culturally competent pain care for marginalized populations. Potential strategies include provider education on biases, enhanced patient-provider communication tools, standardized pain assessment and management protocols, and institutional policies that track and promote equitable pain management practices. Ultimately, addressing these disparities is crucial to improving care quality and outcomes for all patients, regardless of their race, ethnicity, or substance use history.

## Supplementary Information

Below is the link to the electronic supplementary material.

ESM 1 Supplementary file1 (DOCX 34 KB)

## Data Availability

The data can be made available with institutional data use agreements.
