# Supplementary material for: Inequities in Opioid Administration by Race and Ethnicity for Hospitalized Patients With and Without Substance Use Disorders
Source: J Gen Intern Med. 2025 Apr 25;40(12):2944–52. doi: 10.1007/s11606-025-09514-6 (PMC12463787; doi:10.1007/s11606-025-09514-6)
Supplement: Supplementary file 1 — Supplementary file1 (DOCX 34 KB) [file 11606_2025_9514_MOESM1_ESM.docx]

**Supplemental Table 1. Baseline Characteristics for medical patients hospitalized between 2021-2022, No. % (N= 9,102 patients across 13,058 hospitalizations) including all race/ethnicity categories**

| Variable |  | By Race/Ethnicity** | | | |
| --- | --- | --- | --- | --- | --- |
| Selected demographic & hospitalization related variables | White  n= 3,939 | Asian  n =2,110 | Latino  n=1,181 | Black  n= 1,116 | Unknown/Declined  n=63 |
| Age in years, mean (SD) | 63.5 (18.1) | 69.3 (18.6) | 54.7 (19.9) | 58.9 (17.0) | 59.3 (20.1) |
| Length of Stay in days, mean (SD) | 6.5 (11.1) | 6.0 (8.1) | 7.2 (16.9) | 8.3 (18.2) | 9.8 (34.4) |
| Comorbidity Index, mean (SD) | 9.2 (11.2) | 10.9 (11.1) | 9.6 (11.8) | 9.0 (11.2) | 7.3 (9.0) |
| Sex |  |  |  |  |  |
| Female | 1,788 (45.4%) | 1,112 (52.7%) | 633 (53.6%) | 535 (47.9%) | 22 (32.4%) |
| Male | 2,143 (54.5%) | 997 (47.3%) | 548 (46.4%) | 580 (52.0%) | 44 (64.7%) |
| Nonbinary/Other | 8 (0.2%) | 1 (0.1%) | 0 (0.0%) | 1 (0.1%) | 2 (2.9%) |
| Limited English Proficiency |  |  |  |  |  |
| Yes | 211 (5.4%) | 1,100 (52.1%) | 412 (34.9%) | 14 (1.3%) | 16 (23.5%) |
| No | 3,728 (94.6%) | 1,010 (47.9%) | 769 (65.1%) | 1,102 (98.8%) | 52 (76.5%) |
| Substance Use & Pain Related Variables |  |  |  |  |  |
| Presence of Substance Use Disorder | 1,378 (35.0%) | 282 (13.4%) | 347 (29.4%) | 617 (55.3%) | 22 (32.4%) |
| Prescribed MOUD* Prior to Admission | 223 (5.7%) | 31 (1.5%) | 32 (2.7%) | 76 (6.8%) | 1 (1.5%) |
| Prescribed Opioids Prior to Admission | 1,116 (28.3%) | 395 (18.7%) | 333 (28.2%) | 348 (31.2%) | 10 (14.7%) |
| Received Pain or Palliative Care Consultation | 170 (4.3%) | 63 (3.0%) | 50 (4.2%) | 56 (5.0%) | 2 (2.9%) |
| Pain score during hospitalization, mean (SD) | 2.9 (2.5) | 1.6 (2.0) | 2.9 (2.6) | 3.2 (2.6) | 1.8 (2.0) |
| Admitted with moderate/severe pain | 1,192 (30.3%) | 481 (22.8%) | 471 (39.9%) | 452 (40.5%) | 23 (33.8%) |
| Average daily acetaminophen in mg, mean (SD) | 862.7 (950.7) | 670 (846.6) | 933.3 (951.5) | 950.5 (953.7) | 930.2 (1007.6) |
| Average daily ibuprofen in mg, mean (SD) | 53.1 (217.1) | 28.1 (158.9) | 54.5 (231.7) | 51.8 (217.0) | 52.1 (224.2) |
| Variable |  | By Race/Ethnicity** | | | |
| Selected demographic & hospitalization related variables | Multi-Race/Ethnicity  n=242 | Native American or Alaska Native  n=38 | Native Hawaiian or Pacific Islander  n=90 | Southwest Asian or North African  n=126 | Other  n=192 |
| Age in years, mean (SD) | 58.8 (20.8) | 53.9 (14.2) | 61.4 (19.6) | 51.9 (19.7) | 60.5 (19.7) |
| Length of Stay in days, mean (SD) | 6.7 (12.0) | 6.1 (5.7) | 7.8 (26.8) | 4.9 (4.5) | 9.2 (30.4) |
| Comorbidity Index, mean (SD) | 9.1 (11.6) | 12 (12.4) | 8.8 (11.2) | 9.6 (10.2) | 8.3 (11.5) |
| Sex |  |  |  |  |  |
| Female | 120 (49.6%) | 22 (57.9%) | 45 (50.0%) | 62 (49.2%) | 93 (48.4%) |
| Male | 121 (50.0%) | 16 (42.1%) | 45 (50.0%) | 64 (50.8%) | 98 (51.0%) |
| Nonbinary/Other | 1 (0.4%) | 0 (0.0%) | 0 (0.0%) | 0 (0.0%) | 1 (0.5%) |
| Limited English Proficiency |  |  |  |  |  |
| Yes | 38 (15.7%) | 1 (2.6%) | 25 (27.8%) | 38 (30.2%) | 30 (15.6%) |
| No | 204 (84.3%) | 37 (97.4%) | 65 (72.2%) | 88 (69.8%) | 162 (84.4%) |
| Substance Use & Pain Related Variables |  |  |  |  |  |
| Presence of Substance Use Disorder | 79 (32.6%) | 19 (50.0%) | 14 (14.6%) | 32 (25.4%) | 56 (29.2%) |
| Prescribed MOUD* Prior to Admission | 12 (5.0%) | 1 (2.6%) | 2 (2.2%) | 3 (2.4%) | 5 (2.6%) |
| Prescribed Opioids Prior to Admission | 65 (26.9%) | 18 (47.4%) | 13 (14.4%) | 42 (33.3%) | 49 (25.5%) |
| Received Pain or Palliative Care Consultation | 14 (5.8%) | 3 (7.8%) | 3 (3.3%) | 8 (6.4%) | 10 (5.2%) |
| Pain score during hospitalization, mean (SD) | 2.2 (2.3) | 3.2 (2.5) | 1.9 (1.8) | 2.0 (2.0) | 2.2 (2.2) |
| Admitted with moderate/severe pain | 82 (33.9%) | 16 (42.1%) | 29 (32.2%) | 44 (34.9%) | 60 (31.3%) |
| Average daily acetaminophen in mg, mean (SD) | 814.5 (898.0) | 914.1 (1067.8) | 951 3 (997.0) | 954.8 (893.2) | 918.2 (941.6) |
| Average daily ibuprofen in mg, mean (SD) | 46.4 (172.8) | 51.1 (164.4) | 35.8 (153.9) | 52.0 (230.0) | 61.5 (263.8) |

* MOUD: Medication for Opioid Use Disorder

**Supplemental Table 2: ICD-10 codes for the diagnosis of substance use disorders in analysis**

| **Category** | **ICD-10 Codes** |
| --- | --- |
| Alcohol | F10.1, F10.10, F10.12, F10.120, F10.121, F10.129, F10.13, F10.130, F10.131, F10.132, F10.139, F10.14, F10.15, F10.150, F10.151, F10.159, F10.18, F10.180, F10.181, F10.182, F10.188, F10.19, F10.2, F10.20, F10.22, F10.220, F10.221, F10.229, F10.23, F10.230, F10.231, F10.232, F10.239, F10.24, F10.25, F10.250, F10.251, F10.259, F10.26, F10.27, F10.28, F10.280, F10.281, F10.282, F10.288, F10.29, F10.921, F10.93, F10.930, F10.931, F10.932, F10.939, F10.94, F10.95, F10.950, F10.951, F10.959, F10.96, F10.97, F10.98, F10.980, F10.981, F10.982, F10.983, F10.99 |
| Opioids | F11.1, F11.10, F11.12, F11.120, F11.121, F11.122, F11.129, F11.13, F11.14, F11.15, F11.150, F11.151, F11.159, F11.18, F11.180, F11.181, F11.182, F11.188, F11.19, F11.2, F11.20, F11.22, F11.220, F11.221, F11.222, F11.229, F11.23, F11.24, F11.25, F11.250, F11.251, F11.259, F11.28, F11.281, F11.282, F11.288, F11.29, F11.921, F11.922, F11.93, F11.94, F11.95, F11.950, F11.951, F11.959, F11.980, F11.981, F11.982, F11.983, F11.99 |
| Cannabis | F12.1, F12.10, F12.12, F12.120, F12.121, F12.122, F12.129, F12.13, F12.15, F12.150, F12.151, F12.159, F12.18, F12.180, F12.188, F12.2, F12.20, F12.22, F12.220, F12.221, F12.222, F12.229, F12.23, F12.25, F12.250, F12.251, F12.259, F12.28, F12.280, F12.288, F12.29, F12.921, F12.922, F12.93, F12.95, F12.950, F12.951, F12.959, F12.98, F12.980, F12.988, F12.99 |
| Sedative, Hypnotic, or Anxiolytic | F13.1, F13.10, F13.12, F13.120, F13.121, F13.129, F13.13, F13.130, F13.131, F13.132, F13.139, F13.14, F13.15, F13.150, F13.151, F13.159, F13.18, F13.180, F13.181, F13.182, F13.188, F13.19, F13.2, F13.20, F13.22, F13.220, F13.221, F13.229, F13.23, F13.230, F13.231, F13.232, F13.239, F13.24, F13.25, F13.250, F13.251, F13.259, F13.26, F13.27, F13.28, F13.280, F13.281, F13.282, F13.288, F13.29, F13.921, F13.93, F13.930, F13.931, F13.932, F13.939, F13.94, F13.95, F13.950, F13.951, F13.959, F13.96, F13.97, F13.98, F13.980, F13.981, F13.982, F13.988, F13.99 |
| Cocaine | F14.1, F14.10, F14.12, F14.120, F14.121, F14.122, F14.129, F14.13, F14.14, F14.15, F14.150, F14.151, F14.159, F14.18, F14.180, F14.18,1 F14.182, F14.188, F14.19, F14.2, F14.20, F14.22, F14.220, F14.221, F14.222, F14.229, F14.23, F14.24, F14.25, F14.250, F14.251, F14.259, F14.28, F14.280, F14.281, F14.282, F14.288, F14.29, F14.921, F14.922, F14.93, F14.94, F14.95, F14.950, F14.951, F14.959, F14.98, F14.980, F14.981, F14.982, F14.988, F14.99 |
| Stimulants | F15.1, F15.10, F15.12, F15.120, F15.121, F15.122, F15.129, F15.13, F15.14, F15.15, F15.150, F15.151, F15.159, F15.18, F15.180, F15.181, F15.182, F15.188, F15.19, F15.2, F15.20, F15.22, F15.220, F15.221, F15.222, F15.229, F15.23, F15.24, F15.25, F15.250, F15.251, F15.259, F15.28, F15.280, F15.281, F15.282, F15.288, F15.29, F15.921, F15.922, F15.929, F15.93, F15.94, F15.95, F15.950, F15.951, F15.959, F15.98, F15.980, F15.981, F15.982, F15.988, F15.99 |
| Other Hallucinogens and PCP | F16.1, F16.10, F16.12, F16.120, F16.121, F16.122, F16.129, F16.14, F16.15, F16.150, F16.151, F16.159, F16.18, F16.180, F16.183, F16.188, F16.19, F16.2, F16.20, F16.22, F16.220, F16.221, F16.229, F16.24, F16.25, F16.250, F16.251, F16.259, F16.28, F16.280, F16.283, F16.288, F16.29, F16.921, F16.929, F16.94, F16.95, F16.950, F16.951, F16.959, F16.98, F16.980, F16.983, F16.988, F16.99 |
| Nicotine | F17, F17.2, F17.20, F17.200, F17.203, F17.208, F17.209, F17.21, F17.210, F17.213, F17.218, F17.219, F17.22, F17.220, F17.223, F17.228, F17.229, F17.29, F17.290, F17.293, F17.298, F17.299 |
| Inhalant | F18.1, F18.10, F18.12, F18.120, F18.121, F18.129, F18.14, F18.15, F18.150, F18.151, F18.159, F18.17, F18.18, F18.180, F18.188, F18.19, F18.2, F18.20, F18.22, F18.220, F18.221, F18.229, F18.24, F18.25, F18.250, F18.251, F18.259, F18.27, F18.28, F18.280, F18.288, F18.29, F18.921, F18.929, F18.94, F18.95, F18.950, F18.951, F18.959, F18.97, F18.98, F18.980, F18.988, F18.99 |
| Multiple Substances and Other or Unknown | F19.1, F19.10, F19.12, F19.120, F19.121, F19.122, F19.129, F19.13, F19.130, F19.131, F19.132, F19.139, F19.14, F19.15, F19.150, F19.151, F19.159, F19.16, F19.17, F19.18, F19.180, F19.181, F19.182, F19.188, F19.19, F19.2, F19.20, F19.22, F19.220, F19.221, F19.222, F19.229, F19.23, F19.230, F19.231, F19.232, F19.239, F19.24, F19.25, F19.250, F19.251, F19.259, F19.26, F19.27, F19.28, F19.280, F19.281, F19.282, F19.288, F19.29, F19.921, F19.922, F19.929, F19.93, F19.930, F19.931, F19.932, F19.939, F19.94, F19.95, F19.950, F19.951, F19.959, F19.96, F19.97, F19.98, F19.980, F19.981, F19.982, F19.988, F19.99 |

**Supplemental Table 3. Baseline characteristics and Adjusted MME/day^a^ for subgroup without methadone or buprenorphine patients, (N= 8,612 patients across 12,153 hospitalizations)**

| Variable | n (%) | Adjusted MMEs,  Mean (95% CI) | p-value |
| --- | --- | --- | --- |
| Race/Ethnicity |  |  |  |
| White | 3,645 (42.3) | 50.3 (43.7 – 56.8) | ref |
| Asian | 2,082 (24.2) | 30.4 (23.5 – 37.3) | <0.001 |
| Latino | 1,138 (13.2) | 36.8 (30.3 – 43.4) | 0.004 |
| Black or African American | 1,019 (11.8) | 30.9 (25.1 – 36.8) | <0.001 |
| Multi-Race/Ethnicity | 231 (2.7) | 26.5 (18.7 – 34.4) | <0.001 |
| Other | 183 (2.1) | 43.2 (20.7 – 65.8) | 0.570 |
| Southwest Asian or North African | 123 (1.4) | 31.2 (18.1 – 44.3) | 0.028 |
| Native Hawaiian or Pacific Islander | 87 (1.0) | 21.4 (12.3 – 30.4) | <0.001 |
| Unknown/Declined | 66 (0.8) | 32.4 (15.8 – 49.0) | 0.093 |
| Native American or Alaska Native | 38 (0.5) | 28.5 (8.5 – 48.4) | <0.001 |
| Limited English Proficiency |  |  |  |
| Yes | 1,863 (21.6) | 36.9 (29.0 – 44.8) | 0.498 |
| No | 6,749 (78.4) | 39.6 (35.8 – 43.4) |  |
| Sex |  |  |  |
| Male | 4,382 (50.9) | 43.6 (38.0 – 49.2) | ref |
| Female | 4,217 (49.0) | 36.4 (32.4 – 40.4) | 0.012 |
| Nonbinary/Other | 13 (0.2) | 32.5 (8.0 – 57.0) | 0.448 |
| Insurance Status |  |  |  |
| Medicare | 4,614 (53.6) | 38.4 (32.5 – 44.3) | ref |
| Medical | 1,844 (21.4) | 39.7 (33.3 – 46.1) | 0.776 |
| Private/Other | 2,154 (25.0) | 40.2 (33.6 – 46.8) | 0.663 |
| Pain/Palliative Care Consultation |  |  |  |
| Yes | 302 (3.5) | 62.9 (52.0 – 73.8) | <0.001 |
| No | 8,310 (96.5) | 35.0 (31.6 – 38.4) |  |
| Substance Use Disorder Diagnosis |  |  |  |
| Yes | 2,484 (28.8) | 36.9 (32.5 – 41.3) | 0.050 |
| No | 6,128 (71.2) | 43.7 (37.6 – 49.8) |  |
| Prescribed Opioids Prior to Admission |  |  |  |
| Yes | 2,376 (27.6) | 54.9 (49.2 – 60.6) | <0.001 |
| No | 6,236 (72.4) | 18.3 (16.3 – 20.4) |  |
| Admitted with moderate/severe pain |  |  |  |
| Yes | 2,614 (30.4) | 38.9 (35.0 – 42.8) | 0.330 |
| No | 5,998 (69.6) | 41.5 (35.5 – 47.4) |  |
| Continuous Covariates, mean (SD) |  |  |  |
| Age (years) | 63.2 (19.1) | -0.8 (-1.1 - -0.6) | <0.001 |
| Elixhauser comorbidity score | 9.7 (11.2) | 0.3 (0.0 – 0.5) | 0.035 |
| Average acetaminophen/day (mg) | 822.3 (924.0) | 0.01 (0.0 - 0.01) | <0.001 |
| Average ibuprofen/day (mg) | 45.2 (201.2) | -0.02 (-0.02 - -0.01) | <0.001 |
| Average pain score/hospitalization | 1.9 (2.1) | 21.1 (18.1 – 24.2) | <0.001 |
| Length of stay (days) | 6.6 (13.4) | -0.6 (-0.90 - -0.36) | <0.001 |

^a^ adjusted MME/day calculated using average marginal effects. Regression used multivariable negative binomial regression with robust clustering by medical record number.
